# Composition, Production and Procurement of Glass at San Vincenzo al Volturno: An Early Medieval Monastic Complex in Southern Italy

**DOI:** 10.1371/journal.pone.0076479

**Published:** 2013-10-16

**Authors:** Nadine Schibille, Ian C. Freestone

**Affiliations:** 1 Research Laboratory for Archaeology and the History of Art, University of Oxford, Oxford, United Kingdom; 2 Institute of Archaeology, University College London, London, United Kingdom; New York State Museum, United States of America

## Abstract

136 glasses from the ninth-century monastery of San Vincenzo and its workshops have been analysed by electron microprobe in order to situate the assemblage within the first millennium CE glass making tradition. The majority of the glass compositions can be paralleled by Roman glass from the first to third centuries, with very few samples consistent with later compositional groups. Colours for trailed decoration on vessels, for vessel bodies and for sheet glass for windows were largely produced by melting the glass tesserae from old Roman mosaics. Some weakly-coloured transparent glass was obtained by re-melting Roman window glass, while some was produced by melting and mixing of tesserae, excluding the strongly coloured cobalt blues. Our data suggest that to feed the needs of the glass workshop, the bulk of the glass was removed as tesserae and windows from a large Roman building. This is consistent with a historical account according to which the granite columns of the monastic church were spolia from a Roman temple in the region. The purported shortage of natron from Egypt does not appear to explain the dependency of San Vincenzo on old Roman glass. Rather, the absence of contemporary primary glass may reflect the downturn in long-distance trade in the later first millennium C.E., and the role of patronage in the “ritual economy” founded upon donations and gift-giving of the time.

## Introduction

Glass production and technology in early medieval Europe are of interest from a number of perspectives. Closely associated with early monastic foundations [1] and royal palaces such as that of Charlemagne at Paderborn [2], glass was a material largely restricted to the elite, as opposed to the situation in the Byzantine and Islamic eastern Mediterranean where widespread everyday usage as lamps, tableware, drinking vessels and windows continued from the Roman period. Furthermore, as Whitehouse (2003) [3] observed, glass was “a thing that travelled”, produced as a primary material in the tank furnaces of the south-eastern Mediterranean and transported across the world to be re-melted and worked into artefacts [4], [5]. An understanding of fluctuations in the trade of raw glass will add substantially to our understanding of the connectivity of different regions and of the ancient economy. The procurement and use of glass, along with the technologies used and their accessibility, therefore provide evidence for the social, economic and inter-regional relationships during the first millennium CE.

In addition, the glass of the seventh to tenth centuries in Europe occupies the lacuna between the large-scale productions of soda glass of the Roman period and the manufacture of the stained glass windows of the great medieval churches of the Northwest. The introduction of novel, locally-made potash-lime-silica glass and the technologies to produce grisaille and silver stain, along with vivid colours such as translucent ruby red, blue and purple are not well understood. The investigation of early medieval production methods potentially offers insights into issues of innovation and cultural transmission.

The present paper focuses upon the glass from the Monastery of San Vincenzo al Volturno, which is located on the side of the Abruzzi Mountains about equidistant from Rome and Naples and less than 30 km east of the abbey of Monte Cassino. Founded in the early eight century on the site of a late Roman villa, the monastery has yielded substantial evidence for glass working activities from the time of an extensive refurbishment by Abbot Joshua (792–817 CE) in the first decades of the ninth century (for a detailed history of the monastery and its workshops see [6], [7]. Joshua transformed the hitherto modest monastery into one of the great monastic complexes of Carolingian Europe. This included the new church of San Vincenzo Maggiore as well as lodgings for distinguished guests, possibly conceived as a palace for a principal Beneventan donor [8], [9]. Temporary workshops for the production of various building materials (bricks, tiles, metals, glass) were established as part of this rebuilding campaign to the east of the church [10]. The construction of an atrium in this area in the 820s, however, forced the demolition of these workshops and new collective ones were set up about 20 metres further to the south. The new workshops were connected to the *claustrum* by a vaulted passageway and effectively formed an integral part of the monastic city, thus highlighting the significance of this industrial complex [11]. The attack and subsequent burning of the monastery by the Saracens on 10th October 881 provides a firm *terminus ante quem* for the workshop activities if not the monastic community of San Vincenzo at large [7], [12]. This catastrophic event has left us with an exceptionally rich assemblage of relatively well-dated glass from ecclesiastical, domestic and workshop contexts.

Glass technology at San Vincenzo is of interest not only because of the richness of the evidence that it provides about workshop activities. The end of the natron-based or “Roman” glassmaking technology which had dominated the past fifteen hundred years has been dated to the middle of the ninth century [13]. The use of evaporitic sodium carbonate (“natron”) from the lakes of the Wadi el-Natrun in Egypt as a glassmaking flux ceased, and glass began to be made using the ashes of plants and trees. This resulted in soda-lime-silica compositions richer in potassium, magnesium and phosphorus in the South, and potash-lime-silica glasses in the Northwest. San Vincenzo is situated both chronologically and geographically at the crossroads of these developments and offers insights into their effects on regional industries.

**Figure 1:**
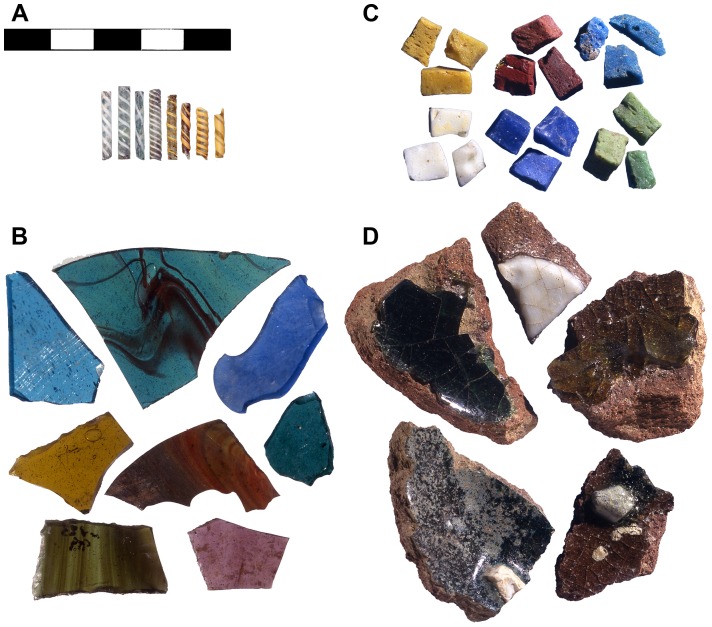
Glass artefacts and working debris from San Vincenzo al Volturno. (A) Bichrome reticella rods with white and yellow threads twisted around a core of colourless glass; (B) Example of bluish-green sheet window glass with red marbling effect; (C) Typical range of mosaic tesserae of various colours found at San Vincenzo; (D) Crucible fragments lined with coloured glass incorporating the remains of marble tesserae.

Excavations conducted within the scope of the San Vincenzo Project directed by Richard Hodges and John Mitchell in the 1980s and 1990s, yielded substantial evidence for the glass working activities that took place there. The glassworkers were responsible for the production of various types of vessels often richly decorated with reticella rods (either plain or bichrome twisted; Fig. 1a) and window panes in a wide range of colours such as purple, red, cobalt blue, turquoise, colourless or green glass with red marbling effects (Fig. 1b) as well as glass imitation gemstones, gilded vessel glass and some remarkable cloisonné enamels [7], [14]–[16]. In excess of 144 glass mosaic tesserae of a great variety of colours were also found at San Vincenzo (Fig. 1c) [17]. Yet, as there is no evidence for the use of decorative mosaics in the monastery and since the tesserae occur only in the workshop area it seems safe to assume that they represent materials to be used as a source of glass and/or colourants rather than for the production of mosaics. No indications of primary glass production that is the manufacture of glass from its raw materials, were found, which means that either glass cullet or ingots must have been imported to San Vincenzo in quite sizeable quantities in order to satisfy the large demands of the monastic refurbishments. Fragments of crucible containing linings of coloured glass are widespread on the site, and some of these appear to contain the remains of marble tesserae which did not melt (Fig. 1d). It seems likely that these represent accidental incorporation of non-glass tesserae by the glassworkers, providing further evidence for the hypothesis that tesserae were used as a raw material on the site.

## Materials and Methods

The studied samples are given in table 1 and include 39 opaque and intensely coloured tesserae (at least 3 examples of each colour identified in the field), 14 reticella canes (plain colourless; plain blue; or yellow or white twisted around a colourless core), 51 vessel fragments made mostly of transparent glass that was colourless or with a bluish or greenish tinge, with some specimens of strong streaky red and cobalt blue, and some 32 flat fragments of sheet, mainly window panes whose colours range from transparent colourless and aqua to translucent deep emerald green, cobalt blue and a deep reddish purple. The glass samples were taken during the San Vincenzo excavations in 1995 by Ian Freestone at the invitation of the excavation director, Prof. Richard Hodges. The mounted samples are currently held by Freestone in the Institute of Archaeology (UCL) in London.

**Table 1:** Compositions of glasses from San Vincenzo in weight percent of oxides (mean of n≥5 measurements per sample), determined by electron microprobe or SEM-EDXA (GAR samples).

Sample no.	colour	SiO_2_	Al_2_O_3_	FeO	CaO	MgO	Na_2_O	K_2_O	TiO_2_	MnO	CoO	CuO	ZnO	SnO_2_	Sb_2_O_5_	PbO	P_2_O_5_	SO_3_	Cl	Comp group
***Reticella rods***																				
SVP-6423–100	white	67.55	2.28	0.46	6.13	1.00	15.60	0.62		0.34					3.74	0.16	0.09	0.39	0.82	
SVP-6423–100	clear	68.58	2.51	0.45	7.01	0.62	16.37	0.86	0.10	1.03		0.13					0.09	0.14	0.79	3
SVP-6423–101	clear	67.80	2.44	0.67	6.86	0.69	16.82	0.95	0.12	1.00		0.09			0.27	0.28	0.11	0.23	0.79	2
SVP-6423–101	white	68.24	2.33	0.32	6.31	0.45	14.41	0.47		0.53				0.10	5.81	0.16	0.10	0.30	0.65	
SVP-6423–102	white	66.91	2.19	0.56	6.06	1.31	15.96	0.85		0.41		0.20			2.61	1.14	0.10	0.32	0.86	
SVP-6423–103	yellow	56.43	1.93	1.41	3.76	0.59	12.69	0.62	0.12	0.75			0.11	0.28	1.44	17.32	0.12	0.18	0.82	
SVP-6423–103	clear	67.23	2.47	0.73	6.59	0.69	16.45	0.91	0.11	0.79		0.32			0.41	0.94	0.12	0.17	0.88	2
SVP-6423–104	clear	66.99	2.17	0.63	6.67	0.69	18.64	0.60	0.14	1.25								0.39	1.09	3
SVP-6423–105	blue	65.43	2.23	1.00	6.73	0.47	14.09	0.60		1.11	0.20	0.25			3.06	2.46	0.16	0.34	0.76	
SVP-6423–106	clear	66.48	2.29	0.67	6.71	0.68	18.24	0.68	0.10	1.25					0.11	0.15	0.07	0.32	0.91	2
SVP-6423–107	yellow	63.16	2.20	1.29	9.66	0.60	13.70	0.50	0.09					0.11	1.07	5.88		0.29	0.96	
SVP-6423–107	clear	66.23	2.46	0.83	7.80	0.82	17.49	1.22	0.11	1.17		0.31				0.38	0.18	0.39	0.81	3
SVP-6423–108	blue	69.07	2.42	0.73	7.28	0.53	14.99	1.00		0.46	0.09	0.20			1.88	0.41	0.14	0.29	0.75	
SVP-6423–109	white	66.97	2.18	0.52	6.64	1.49	16.00	0.60	0.07	0.18					4.21	0.17	0.12	0.34	0.79	
SVP-6423–109	clear	68.89	2.44	0.44	7.46	0.59	16.83	0.71	0.10	0.98						0.11	0.10	0.23	1.07	3
SVP-6423–110	yellow	63.54	2.04	1.31	5.81	0.41	14.38	0.50	0.10					0.09	3.11	6.33		0.27	1.03	
SVP-6423–110	clear	68.52	2.45	0.40	7.04	0.56	16.76	0.89	0.08	0.76							0.12	0.22	1.00	2
SVP-6423–111	white	68.11	2.34	0.35	6.77	0.56	14.60	0.53		0.39					4.72	0.15	0.13	0.37	0.65	
SVP-6423–112	yellow	65.70	1.93	0.93	5.61	0.44	15.11	0.50	0.11					0.11	0.80	6.10		0.27	1.06	
SVP-6423–112	clear	67.91	2.28	0.58	7.50	0.67	17.62	0.84	0.12	0.98							0.11	0.29	0.92	3
SVP-6423–113	clear	67.89	2.55	0.62	7.28	0.68	16.73	0.89	0.12	0.92		0.30			0.23	0.42	0.14	0.25	0.96	2
***Mosaic Tesserae***																				
SVP-6423–114a	blue light	68.09	2.42	0.59	6.67	0.60	13.79	0.80	0.09	0.57					4.99	0.14	0.22	0.46	0.59	
SVP-6423–114b	blue light	67.25	2.42	0.77	5.77	0.57	15.69	0.63	0.13	0.41					4.30	0.34	0.11	0.48	0.69	
SVP-6423–114c	blue light	68.54	2.34	0.51	6.19	0.48	15.31	0.56	0.09	0.43					3.54		0.09	0.48	0.73	
SVP-6423–115a	white	67.06	2.46	0.39	6.84	0.54	14.17	0.68		0.52					5.01		0.16	0.41	0.71	
SVP-6423–115b	white	65.97	1.79	0.36	6.53	3.83	16.91	0.55	0.09						2.79			0.28	0.86	
SVP-6423–115c	white	65.80	1.97	0.84	5.45	2.17	17.59	0.50	0.16						4.77	0.11		0.32	0.90	
SVP-6423–116a	yellow	66.66	1.94	0.68	6.49	0.56	17.35	0.59	0.11						0.64	2.78	0.07	0.33	1.23	
SVP-6423–116b	yellow	63.62	2.11	0.98	5.71	0.56	16.64	0.52	0.20	0.38					0.63	5.94	0.08	0.30	1.28	
SVP-6423–116c	yellow	65.74	2.18	0.62	5.02	0.55	15.78	0.59	0.15	0.32					0.88	4.94	0.07	0.29	1.14	
SVP-6423–117a	turquoise	67.71	2.09	0.54	5.63	0.53	17.35	0.64	0.09	0.28		1.95		0.11	1.34	0.15	0.08	0.29	1.19	
SVP-6423–117b	turquoise	66.69	2.10	0.45	5.81	0.49	17.42	0.61	0.07	0.28		2.96		0.23	0.93	0.31	0.10	0.30	1.06	
SVP-6423–117c	turquoise	65.97	2.27	0.60	6.86	0.47	14.80	0.62		0.41		2.41		0.15	4.18	0.18	0.15	0.42	0.74	
SVP-6423–117d	turquoise	66.32	2.19	0.65	4.24	0.51	17.81	0.70	0.19			2.57	0.18	0.24	2.34	0.19		0.23	1.41	
SVP-6423–118a*	mid-blue	70.57	2.52	0.58	6.09	0.43	15.77	0.64	0.07	0.71	0.05	0.09			1.13	0.21	0.14	0.12	1.03	
SVP-6423–118b*	mid-blue	69.11	2.16	0.91	4.90	0.61	18.38	0.53	0.12	0.35	0.03	0.26			1.08	0.10	0.08	0.17	1.22	
SVP-6423–118c*	mid-blue	69.50	2.08	0.59	5.37	0.44	17.99	0.61	0.10	0.34	0.03	0.05			1.11	0.19	0.07	0.18	1.15	
SVP-6423–119a*	deep blue	69.24	2.40	0.75	6.57	0.41	14.72	0.57	0.07	0.41	0.16	0.17			3.66		0.13	0.22	0.69	
SVP-6423–119b*	deep blue	69.05	2.66	0.86	8.22	0.53	13.97	0.51	0.07	0.20	0.09	0.12			2.68		0.14	0.19	0.72	
SVP-6423–119c*	deep blue	69.37	2.39	0.74	6.52	0.44	14.74	0.61		0.43	0.16	0.15			3.63		0.14	0.18	0.70	
SVP-6423–120a	blue/grey	69.47	2.10	0.35	6.16	0.45	16.60	0.53		0.32					2.17		0.07	0.41	0.95	
SVP-6423–120b	blue/grey	69.55	2.27	0.58	7.05	0.54	16.15	0.67	0.10	0.66					1.03		0.14	0.34	1.08	
SVP-6423–120c	blue/grey	66.78	2.65	0.73	6.30	0.57	16.00	0.73	0.11	0.44					3.44	0.19	0.13	0.41	0.80	
SVP-6423–120d	blue/grey	66.17	2.52	0.67	6.21	0.59	15.93	0.74	0.11	0.42					4.50	0.26	0.12	0.40	0.78	
SVP-6423–121a	turquoise	67.43	2.06	0.35	6.34	0.50	17.71	0.57	0.07	0.55		1.63		0.16	0.67	0.47	0.07	0.27	1.20	
SVP-6423–121b	turquoise	68.27	2.20	0.56	5.93	0.55	17.12	0.60	0.07	0.40		2.05		0.12	0.80	0.14	0.09	0.31	1.22	
SVP-6423–121c	turquoise	68.92	1.97	0.46	5.63	0.47	18.35	0.52	0.09	0.27		1.07		0.10	0.55			0.30	1.34	
SVP-6423–122a	red	63.05	2.12	1.90	7.07	2.05	16.14	1.37	0.18	0.65		1.77		0.20		1.08	0.72	0.25	1.01	
SVP-6423–122b	red	56.70	1.59	1.33	8.73	2.27	12.24	2.61	0.16	0.39		2.21		0.30		7.95	0.95	0.16	0.93	
SVP-6423–122c	red	63.11	2.40	2.07	7.28	1.26	15.63	1.21	0.15	0.71		1.31		0.19	0.26	2.47	0.49	0.27	0.97	
SVP-6423–123a	black p.	69.34	2.30	0.10	7.98	0.54	15.27	0.48		2.42							0.15	0.22	1.05	
SVP-6423–123b	black gr.	67.30	2.42	5.06	6.94	0.45	15.52	0.68	0.08	0.52						0.13	0.12	0.17	1.11	
SVP-6423–123c	black gr.	67.04	2.42	5.15	6.87	0.47	15.77	0.70	0.08	0.45							0.15	0.17	1.00	
SVP-6423-124a	green	66.59	2.33	0.59	6.79	0.55	16.50	0.80	0.10	0.35		1.64		0.17	0.25	1.36	0.13	0.26	1.08	
SVP-6423–124b	green	67.97	2.10	0.40	6.51	0.52	17.62	0.59	0.08	0.62		1.32			0.35	0.47	0.11	0.30	1.33	
SVP-6423–124c	green	68.40	2.22	0.43	6.83	0.55	16.98	0.63	0.09	0.75		1.04			0.21	0.56	0.16	0.25	1.24	
SVP-6423–124d	green	66.98	2.58	0.63	6.58	0.55	17.13	0.83	0.11	0.51		1.48	0.15		0.29	0.98	0.12	0.35	1.19	
SVP-6423–125a	green	67.37	2.36	0.50	7.25	0.50	15.45	0.70	0.09	0.76		1.51		0.15		1.93	0.15	0.22	1.04	
SVP-6423–125b	green	66.17	2.46	0.57	6.85	0.55	14.78	0.85	0.12	0.74		1.04	0.21		0.25	3.87	0.15	0.20	0.95	
SVP-6423–125c	green	66.92	2.18	0.64	6.96	0.54	15.07	0.69	0.09	0.49		1.53	0.10	0.20	0.12	2.78	0.15	0.21	1.06	
***Vessels***																				
SVP-v–44738	blue	66.12	2.48	0.97	7.28	0.64	15.17	1.28	0.07	0.64	0.09	0.72			1.83	1.10	0.17	0.30	0.83	
SVP-v–44739	blue	68.68	2.43	0.83	8.02	0.49	17.79	0.59	0.08	0.21	0.06					0.09	0.08	0.30	1.02	
SVP-v–44740	blue	67.26	2.51	0.99	8.11	0.59	17.06	0.78	0.09	0.61	0.07	0.24			0.65	0.20	0.18	0.23	0.97	
SVP-v–44742	blue	67.64	2.44	0.90	7.28	0.62	16.03	0.95	0.11	0.59	0.07	0.32			1.62	0.54	0.14	0.35	0.82	
SVP-v–44744	blue	68.93	2.34	0.72	7.46	0.49	15.07	0.63	0.07	0.55	0.06				1.91	0.47	0.15	0.29	0.84	
SVP-v–44745	blue	68.16	2.05	1.86	5.89	0.62	18.63	0.60	0.10		0.03	0.46			0.47	0.09	0.10	0.37	1.26	
SVP-v–44765	blue	67.56	2.47	0.65	6.95	0.59	15.89	0.95	0.10	0.48	0.04	0.11			2.27		0.16	0.42	0.58	
SVP-v–44766	blue	67.71	2.35	0.83	7.59	0.57	15.53	0.75	0.07	0.52	0.10				1.95	0.22	0.17	0.37	0.75	
SVP-v–6423.11-ST	blue	67.53	2.45	0.81	7.06	0.55	16.17	0.64	0.07	0.55	0.08	0.19			1.28	0.28	0.20	0.26	0.86	
SVP-v–6423.17-ST	blue	66.70	2.61	0.84	7.14	0.58	16.04	1.04	0.08	0.58	0.05	0.33			1.29	0.38	0.20	0.21	0.64	
SVP-v–6423.7-ST	blue	67.80	2.47	0.70	7.36	0.51	15.31	0.80	0.08	0.63	0.07	0.13			1.81	0.33	0.21	0.30	0.74	
SVP-v–6423.8-ST	blue	66.78	2.51	0.85	7.24	0.56	15.76	0.86	0.08	0.53	0.06	0.38			1.49	0.50	0.21	0.23	0.72	
SVP-v–6423.9-ST	blue	67.27	2.48	0.87	7.41	0.56	15.89	0.90	0.07	0.58	0.07	0.25			1.41	0.33	0.22	0.29	0.63	
SVP-v–44753	colourless	66.75	2.44	0.75	7.22	0.70	16.25	1.07	0.11	0.60		0.71			1.04	0.93	0.17	0.28	0.77	1
SVP-v–44754	colourless	67.30	2.40	0.80	7.07	0.68	16.17	1.13	0.11	0.64		0.70		0.10	0.97	0.83	0.17	0.35	0.65	1
SVP-v–44755	colourless	66.39	2.45	0.83	6.86	0.81	16.59	1.16	0.13	0.65	0.08	0.82			0.73	1.03	0.19	0.33	0.72	1
SVP-v–44756	colourless	65.91	2.41	0.88	6.87	0.83	16.32	1.09	0.08	0.60		0.85			0.65	1.73	0.22	0.30	0.81	1
SVP-v–44757	colourless	66.48	2.37	0.85	6.57	0.75	16.81	0.99	0.11	0.56		1.20		0.10	0.66	1.23	0.15	0.30	0.91	1
SVP-v–6423.20-ST	colourless	65.93	2.51	0.72	6.50	0.64	16.89	0.78	0.11	0.46		1.12		0.10	0.82	0.89	0.21	0.30	0.89	1
SVP-v–44732	colourless	68.04	2.33	0.56	7.51	0.68	16.89	0.90		1.06		0.15			0.16	0.40	0.14	0.25	0.93	2
SVP-v–44733	colourless	68.26	2.43	0.64	7.18	0.69	16.95	0.96	0.11	0.90		0.13				0.36	0.13	0.24	0.71	2
SVP-v–44734	colourless	67.63	2.67	0.80	6.89	0.76	17.00	0.75	0.20	1.18					0.11	0.21	0.09	0.22	1.03	2
SVP-v–44736	colourless	69.93	1.97	0.42	6.32	0.59	17.48	0.91	0.10	0.25					0.46	0.09	0.07	0.24	1.17	2
SVP-v–44758	colourless	68.18	1.80	0.29	5.91	0.42	19.57	0.41							0.96		0.05	0.31	1.60	2
SVP-v–44759	colourless	68.73	2.25	0.40	6.75	0.55	17.53	0.74	0.12	0.65					0.21	0.15	0.12	0.22	1.17	2
SVP-v–6423.13-ST	colourless	66.83	2.48	0.64	7.29	0.67	16.89	0.98	0.12	0.76		0.13			0.29	0.21	0.23	0.23	0.88	2
SVP-v–6423.14-ST	colourless	66.86	2.55	0.65	7.55	0.69	16.92	0.98	0.09	0.74		0.14			0.24	0.19	0.34	0.22	0.85	2
SVP-v–6423.15-ST	colourless	66.98	2.55	0.69	7.38	0.68	16.65	1.08	0.12	0.76		0.25			0.37	0.41	0.21	0.25	0.78	2
SVP-v–6423.16-ST	colourless	67.08	2.41	0.62	7.19	0.59	16.55	1.84	0.07	0.70		0.13			0.29	0.24	0.15	0.24	0.94	2
SVP-v–6423.18-ST	colourless	67.04	2.51	0.66	7.23	0.66	16.91	0.98	0.10	0.81		0.11			0.21	0.18	0.18	0.25	0.92	2
SVP-v–6423.19-ST	colourless	67.16	2.56	0.65	7.34	0.64	16.59	1.07	0.12	0.72		0.18			0.27	0.29	0.19	0.24	0.90	2
SVP-v–6423.22-ST	colourless	66.66	2.47	0.64	7.41	0.64	15.90	2.40	0.12	0.75		0.16			0.30	0.29	0.21	0.26	0.81	2
SVP-v–6423.23-ST	colourless	66.98	2.51	0.66	7.44	0.67	16.04	2.21	0.12	0.74		0.15			0.28	0.32	0.17	0.26	0.81	2
SVP-v–6423.25-ST	colourless	67.17	2.20	0.50	6.96	0.54	17.79	0.58	0.09	0.85					0.16	0.49	0.13	0.31	1.05	2
SVP-v–44735	colourless	69.73	2.33	0.22	7.63	0.49	16.00	0.45		1.42							0.14	0.19	1.13	3
SVP-v–44737	colourless	68.48	2.57	0.57	7.58	0.62	16.47	1.05	0.08	1.06						0.19	0.12	0.26	0.90	3
SVP-v–44761	colourless	66.87	2.24	0.63	7.20	0.70	18.64	0.71	0.14	1.11						0.10	0.11	0.41	0.97	3
SVP-v–44762	colourless	66.32	2.28	0.57	7.25	0.65	16.27	2.52	0.12	1.30						0.09	0.10	0.32	1.01	3
SVP-v–6423.24-ST	colourless	67.80	2.38	0.47	8.01	0.69	16.04	0.67	0.08	1.64							0.34	0.16	1.05	3
SVP-v–44741-GAR	colourless	68.3	2.1	0.7	7.1	2.5	13.2	2.1	0.1	2.5							0.3	0.3	0.9	plant ash?
SVP-v–44760-GAR	colourless	68.9	2.5	0.5	6.9	0.7	17.6	0.8	0.1	0.7								0.4	0.9	
SVP-v–44743-GAR	greenish	69.2	2.4	0.7	10.1	0.6	15.3	0.2	0.3									0.3	0.9	Egypt 2
SVP-v–44752-GAR	greenish	66.5	2.4	0.7	6.2	0.9	17.8	0.8	0.1	0.4		1.2			1.0	0.7		0.5	0.9	1
SVP-v–44768	greenish blue	66.18	2.39	0.80	7.01	0.68	16.56	0.88	0.12	0.53		0.92			0.92	1.37	0.13	0.34	0.93	1
SVP-v–6423.10-ST	greenish blue	65.81	2.62	0.86	7.31	0.67	16.40	1.21	0.10	0.56		0.64		0.28	0.87	0.57	0.22	0.21	0.64	1
SVP-v–6423.12-ST	greenish blue	65.43	2.57	0.84	6.91	0.64	16.68	1.14	0.11	0.45		1.42	0.12	0.09	0.89	0.93	0.25	0.25	0.59	1
SVP-v–44748-ST	greenish blue	65.66	2.43	0.89	7.15	1.1	17.77	0.85	0.08	1.15		0.25			0.53	0.34	0.16	0.54	0.8	2
SVP-v–44764	pinkish	66.85	2.41	0.68	7.24	0.68	19.05	0.76	0.11	1.17							0.11	0.40	0.84	3
SVP-v–44751	purple	67.68	2.40	0.22	7.64	0.55	18.11	0.61	0.08	1.61							0.07	0.31	1.08	
SVP-v–44731	red	62.50	2.43	1.49	6.88	0.99	15.30	1.13	0.11	0.60				0.20	0.29	4.96	0.26	0.33	0.81	
SVP-v–44731b	red	66.78	2.54	1.01	7.24	0.77	16.02	1.20	0.11	0.71		0.81		0.09	0.17	0.99	0.17	0.30	0.68	
SVP-v–44767	red transl.	67.16	2.44	0.57	7.49	0.64	16.73	1.00	0.10	0.78		1.48	0.61			0.18	0.11	0.19	0.83	
***Windows/sheets***																				
SVP-w–44697P*	blue	68.66	2.36	0.84	7.20	0.53	15.46	0.67	0.08	0.47	0.08	0.13			1.40	0.17	0.13	0.22	0.79	
SVP-w–44715P*	blue	67.79	2.32	0.70	6.85	0.54	15.81	0.80	0.08	0.54	0.04	0.52			1.64	0.61	0.14	0.24	0.82	
SVP-w–44719S*	blue	68.52	2.24	0.78	6.63	0.85	15.69	0.90	0.08	0.43	0.05	0.20			1.92	0.21	0.12	0.19	0.50	
SVP-w–44723P*	blue	68.72	2.32	0.77	6.95	0.59	15.18	0.67	0.07	0.53	0.07	0.20			2.06	0.27	0.15	0.23	0.65	
SVP-w–44725W*	blue	68.32	2.38	0.72	7.41	0.55	15.55	1.00	0.09	0.53	0.04	0.51			1.53	0.40	0.14	0.22	0.52	
SVP-w–44729	blue	68.00	2.27	0.83	7.53	0.85	15.74	0.64	0.11	0.55	0.08	0.31			1.90	0.25	0.15	0.31	0.54	
SVP-w–44700S*	colourless	68.30	2.85	0.80	6.03	0.75	15.20	0.94	0.12	0.43		0.50		0.10	0.63	1.75	0.17	0.17	0.75	1
SVP-w–44701-GAR	colourless pale gr.	66.3	2.6	0.9	6.8	0.8	17.3	1.0	0.1	0.7		1.2		0.3	0.9	1.0	0.1	0.3	0.8	1
SVP-w–44714-GAR	colourless	67.2	2.6	0.8	7.1	0.7	17.0	1.0	0.1	0.7		0.3			0.4	0.2		0.4	0.7	2
SVP-w–44724Y*	colourless	68.80	2.37	0.65	6.57	0.62	16.60	0.89	0.10	0.61		0.24			0.44	0.28	0.15	0.16	0.89	2
SVP-w–44708Y*	colourless	70.69	2.82	0.44	6.38	0.57	15.43	0.52	0.07	1.10							0.09		1.25	3
SVP-w–44726	colourless	66.78	2.39	0.66	7.36	0.75	18.21	0.89	0.15	1.13		0.09				0.15	0.10	0.41	0.79	3
SVP-w–44720	deep purple red	67.39	2.18	0.64	7.30	1.05	17.67	0.72	0.12	0.80		0.22	0.12		0.14	0.17	0.12	0.27	0.75	
SVP-w–44728	deep purple red	64.91	3.15	1.24	6.15	1.84	16.75	2.44	0.20	1.59							0.38	0.13	1.07	plant ash?
SVP-w–44698	green	54.69	2.19	1.09	6.29	0.91	13.34	1.00	0.10	0.33		4.97	0.14	0.52	0.76	11.96	0.22	0.30	0.52	
SVP-w–44699	green	59.92	2.13	0.98	6.46	0.76	13.91	1.04	0.08	0.36		4.15	0.11	0.31	0.89	7.57	0.21	0.33	0.62	
SVP-w–44713	green	65.65	2.32	1.68	5.06	2.65	15.97	0.77	0.12	0.36		0.92	0.13	0.12	0.91	1.14	0.24	0.26	0.88	
SVP-w–44718	green	65.10	2.26	0.74	6.39	0.82	16.03	0.97	0.07	0.34		5.04		0.10	0.86	0.68	0.12	0.30	0.67	
SVP-w–44721	green	67.14	2.17	0.65	7.65	1.39	17.44	0.76	0.13	0.90		0.29			0.23	0.13	0.15	0.31	0.65	
SVP-w–44713	green/red	64.73	2.23	0.81	6.36	0.76	16.84	0.69	0.13	0.38		2.59	0.10	0.12	0.96	1.09	0.19	0.30	0.84	
SVP-w–44705	olive	64.10	2.12	6.53	6.55	0.76	16.05	0.88	0.08	0.68					0.23	0.10	0.12	0.22	0.99	
SVP-w–44730	olive dark	67.60	2.29	0.86	6.95	1.64	17.15	0.87	0.10	0.67		0.17		0.10	0.73	0.18	0.23	0.18	0.60	
SVP-w–44704	pale blue/green	65.92	2.37	0.76	6.98	0.70	16.54	0.75	0.10	0.64		0.76		0.11	1.11	1.33	0.20	0.30	0.97	
SVP-w–44707	pale blue/green	65.97	2.43	0.82	7.08	0.78	16.24	0.94	0.11	0.67		0.74		0.10	0.87	1.48	0.18	0.35	1.02	1
SVP-w–44710-GAR	pale blue/green	67.7	2.5	0.7	7.0	0.9	17.3	0.8	0.1	0.7		0.6			0.8	1.0	0.2	0.5	0.9	1
SVP-w–44711	pale blue/green	66.14	2.51	0.73	6.73	0.63	15.28	0.80	0.09	0.56		0.92		0.11	1.69	2.40	0.14	0.38	0.82	1
SVP-w–44712	pale blue/green	66.23	2.27	0.79	6.45	0.75	16.47	0.73	0.13	0.40		2.32		0.14	0.67	1.16	0.17	0.37	0.90	1
SVP-w–44716-GAR	pale blue/green	65.1	2.8	0.8	6.3	0.8	17.1	0.9		0.6		0.5			1.0	2.5		0.5	0.7	1
SVP-w–44727-GAR	pale blue/green	67.3	2.5	0.7	6.4	0.7	18.1	0.9	0.1	0.4		0.7			1.1	0.3		0.4	0.7	1
SVP-w–44706	pale blue/turquoise	64.98	2.18	0.77	6.21	0.85	16.88	0.72	0.09	0.36		3.63		0.13	0.76	1.07	0.16	0.33	0.90	1
SVP-w–44709-GAR	pale blue/green	65.5	2.6	0.8	6.7	0.8	17.0	0.7	0.1	0.7		0.2		2.7	0.7	2.4		0.4	0.8	2
SVP-w–44702	red streaky	63.73	2.76	1.05	7.03	0.74	16.17	1.34	0.09	0.82		2.32	0.67	0.34	0.86	1.17	0.19	0.28	0.59	
SVP-w–44702	red streaky	66.00	2.45	0.84	7.14	0.73	16.47	1.25	0.11	0.72		0.91		0.11	0.76	1.04	0.18	0.20	0.66	

Resin-mounted, ground, polished and carbon-coated sections of the individual glass samples (about 1–2 mm^3^) were analysed for 22 elements (Na, K, Mg, Ca, Ba, Ti, Cr, Mn, Fe, Co, Ni, Cu, Zn, Al, Si, Sn, Pb, P, As, Sb, S, Cl) using a JEOL 8600 electron microprobe with four wavelength-dispersive spectrometers (WDS) (for details see [18]). Raw elemental concentrations were converted into weight percent (wt%) oxide values using a PAP correction programme and the mean of at least 5 measurements per sample (n≥5) are given in Table 1. The relative standard deviation (RSD) of the repeated measurements was within 1% for SiO_2_ and Na_2_O, about 3–4% for CaO, MgO, K_2_O and Al_2_O_3_ and between 8% and 10% for TiO_2_, MnO, Fe_2_O_3_ and CuO. A small sub-group of 14 samples (marked with an asterisk (*) in Table 1) were analysed using a JEOL 8100 microprobe with 10 spots per sample to optimize precision on colourant elements Co and Sb, yielding RSDs typically better than 20% and 10% respectively. A set of 10 glasses analysed by SEM-EDXA (technique of [5]) were also included, and are indicated by the suffix GAR (Table 1), along with a series of vessels analysed using a JEOL JXA 8600 microprobe, indicated by the suffix ST (technique of [19]). The correspondence between our measurements and the expected compositions of Corning Museum Ancient Glass Standards [20] were, for all methods, generally within a few percent relative and no corrections to our measurements were made. Graphical comparisons of data are based upon either the uncorrected data as analysed, or the so-called “reduced composition” where the colourant additives have been removed and the composition of the base glass re-calculated to 100%, based upon the oxides of Si, Al, Ca, Mg, Na and K [20].

## Results

### General

**Figure 2:**
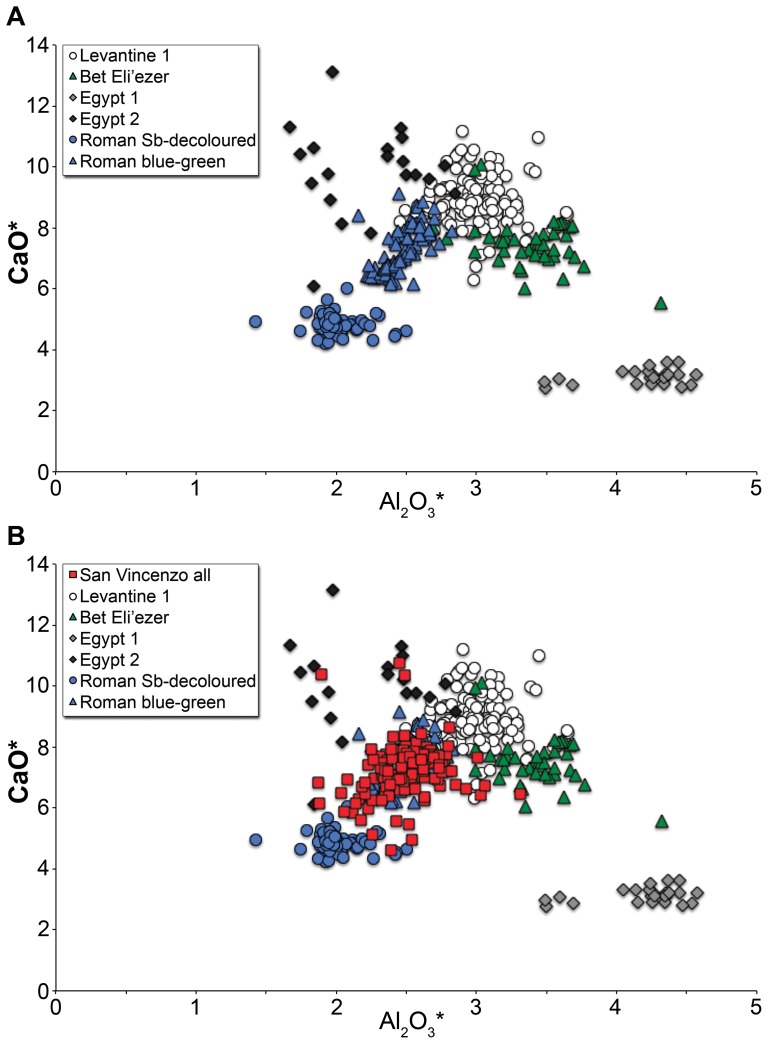
Lime and alumina contents of glass from San Vincenzo compared with those of established first millennium production groups. (A) Primary production groups of the fourth to ninth centuries (sources of data given in [65], [66] compared with typical blue-green [61] and antimony-decolourised [67] Roman glass of the first to third centuries; (B) All glass from San Vincenzo compared with the major glass groups show strong similarities to Roman blue-green glass (reduced, normalised data).

All samples analysed are soda-lime-silica glasses with approximately 68% SiO_2_, 7% CaO and 16% Na_2_O. The average Al_2_O_3_ concentration is about 2.5%, while MgO and K_2_O are typically in the range of 0.7% and 0.9%, respectively. Hence the assemblage consists predominantly of typical natron glasses of the first millennium CE [21], [22]. A few exceptions, with higher MgO and/or K_2_O are discussed below. Lime-alumina plots are routinely used to compare major natron glass groups, as they reflect the compositions of the glassmaking sands and separate geographical and chronological variants [5]. All of the glasses from San Vincenzo analysed here are compared with the compositions of some of the major fourth- to ninth- century natron glass groups in addition to Roman green-blue and colourless transparent glasses of the first to third centuries in Fig. 2. It is observed that the San Vincenzo glasses are close to the earlier Roman glasses and not, as might have been expected, to the later material.

### Mosaic Tesserae

**Figure 3:**
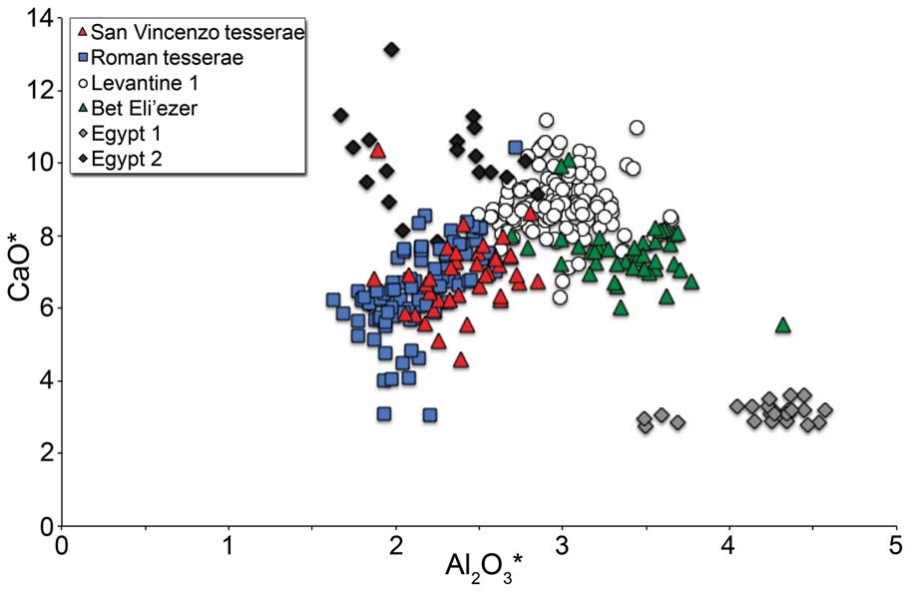
Mosaic tesserae from San Vincenzo compared to Roman tesserae. 39 mosaic tesserae of different colours from San Vincenzo compared to 95 glass tesserae from first- to third-century mosaics from Italy and North Africa in terms of their lime and alumina concentrations (Roman sample excludes opaque reds; Freestone unpublished data).

The tesserae are all natron-type glasses, except some of the white tesserae that have higher MgO, and the red tesserae that have elevated K_2_O and MgO (Table 1). The reduced compositions are typical of Roman opaque glasses, including tesserae, of the first to third centuries CE [23]–[27] (Fig. 3). Colourants and opacifiers include cobalt (blue), copper (turquoise, green and red), calcium antimonate (white), lead antimonate (yellow), manganese (purple, black) and iron (green, black). With the exception of the red tesserae, which are opacified by sub-micrometer copper-rich particles, probably copper metal [28], the opacifying phases in the tesserae are typically calcium or lead antimonate particles. No evidence of tin-oxide compounds, the characteristic opacifiers in opaque glass from the fourth century onwards [29], was found. Tin concentrations are above background levels in some tesserae but at a few tenths of one percent or lower; these are associated with elevated copper or lead and reflect the use of scrap metals such as bronze and pewter as sources of colourants (e.g. [23], [30]. A single analysed sample has elevated tin oxide. This sample, however, is not a tessera but a weakly-coloured sheet (#44709, Table 1) and is not opaque.

Two of the white tesserae have MgO levels above 2%, but without accompanying high K_2_O. Therefore, although not typical natron-type compositions, these are unlikely to represent the use of plant ash. Opaque white glasses with values of MgO above one per cent are known from other Roman glasses, including mosaics [24], [26], [31] but the significance of the elevated magnesium is not well understood. In the present case, the elevated magnesia emphasises the general similarity of the San Vincenzo tesserae to Roman glass of the first half of the first millennium. Similarly, opaque red glass with elevated magnesia and potash occurs widely and is commonly considered to represent the use of a raw glass made with a plant ash-based source of soda [31], [32]. However, it is noted that these glasses are intermediate between natron and typical plant ash glasses; in only one of the three examples analysed here are MgO and K_2_O both above 2%. It seems more likely that an ash-bearing component has been added to a natron-based glass, probably charcoal-containing fuel ash to promote the reduction of the copper to metal [33].

Overall, the compositions of the tesserae found at San Vincenzo correspond well to those of Roman opaque tesserae of the first to third centuries, with no evidence of the tin-opacification known from the succeeding period. This is fully consistent with the archaeological evidence which suggests that the tesserae were being used as raw materials for glass working.

### Coloured glass used in vessels

Opaque white and yellow glasses are found in vessels in the form of decorative thread-like trails; these were applied as thin filigree glass canes, fragments of which were recovered in considerable quantities from the site (Fig. 1a). Compositions of the opaque glasses from the canes are conformable with the tesserae, including elevated MgO in some of the whites, although one of the yellows from the canes has significantly higher lead at 17%, as opposed to 5–6% for most of those from the tesserae and the other canes (Table 1). However, Roman opaque yellow glasses are known to have a wide range of lead contents [26] and a single mosaic may contain yellow tesserae with similarly disparate lead contents (e.g. [32]). Blue canes have compositions similar to those of the opaque deep-blue tesserae, with CoO around 0.2%. Overall, the coloured glasses in the reticella canes are similar to those of the tesserae and fully consistent with colours derived from earlier Roman glass. While not every glass composition we have measured in the canes is replicated completely by the composition of an analysed tessera, there are sufficient parallels to suggest that this is the effect of the small sample number, rather than some fundamental difference between the two groups.

### Translucent strongly coloured window and vessel glass

Translucent coloured glass occurs in the form of sheets for windows and other ornamental items as well as vessels. The most common colour is blue. Blue translucent sheet and vessel glass typically contains around 0.1% CoO as colourant, which is typical for most ancient soda-lime-silica glass. However, it also contains relatively high concentrations of Sb_2_O_5_, at 1.5–2.0%, levels which typically characterise the use of antimony as an opacifier, although these glasses are translucent.

**Figure 4:**
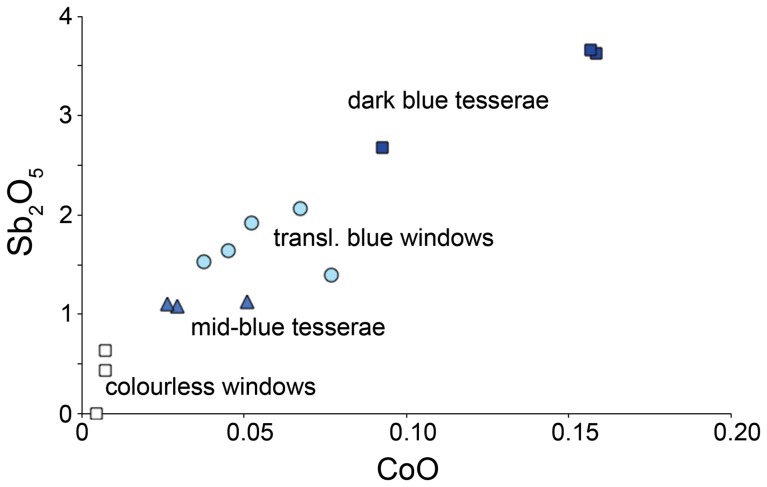
Blue glasses from San Vincenzo. Cobalt and antimony oxide contents of selected blue tesserae (dark and mid-blue), deep blue translucent windows and weakly coloured transparent window glass, showing that blue window glass may be explained as a mixture of mid- and dark-blue mosaic tesserae.

To understand the production of translucent blue glass, we conducted high precision analyses (described above) for fourteen glasses, to improve the cobalt and antimony measurements. Results are summarised in Fig. 4, and indicate that the blue windows could have been formed by remelting a mixture of mid- and dark-blue tesserae. Rapid cooling and/or an absence of heat-treatment would have precluded the recrystallization of the calcium antimonate opacifying phase. Confirmation of this model was found by SEM examination of the blue windows, which sometimes contain sparse calcium antimonate particles and macroscopically appeared somewhat cloudy.

Other translucent colours used in window sheets and vessels and consistent with a mixture of tesserae include streaky reds, apparently a mixture of opaque red tesserae and paler colours. As expected, these samples have elevated iron, copper and lead oxides. Production of clear copper red glass is challenging due to the need to control the size and density of copper metal particles in the glass; early medieval glass workers tried to circumvent this problem by producing a glass with thin streaks of opaque red in a pale matrix. A small number of window sheets, notably an emerald green type containing around 5% copper oxide and varying concentrations of lead, do not correspond to analysed tesserae. A single analysed example of a manganese red-purple (sheet #44728) has elevated MgO (1.84%) and K_2_O (2.44%) and is the only sample likely to have been made (formed) at San Vincenzo that we consider to be a potential plant ash glass. However, the MgO level is marginal and in some respects this resembles an example of the widespread fourth century glass type HIMT (high iron, manganese and titanium) which has been contaminated by potassium; its origin is considered unclear.

### Weakly coloured and colourless transparent glass

Colourless and pale blue-green glasses are found in the forms of the transparent element of reticella canes, window sheets and vessels. With one exception (#44741-GAR), a vessel fragment that was selected for analysis due to its unusual corrosion characteristics, all these samples have low MgO and K_2_O and were therefore produced using a mineral soda (natron) as flux. MnO and Sb_2_O_5_, which were used as decolourants in glass during the first millennium CE, are frequently present up to just over 1%. However, the ranges of CuO and PbO are wider than normally encountered, ranging up to 1.4% and 1.8% respectively. Usually, these oxides are present only at trace levels in colourless or weakly-coloured natron glass.

**Figure 5:**
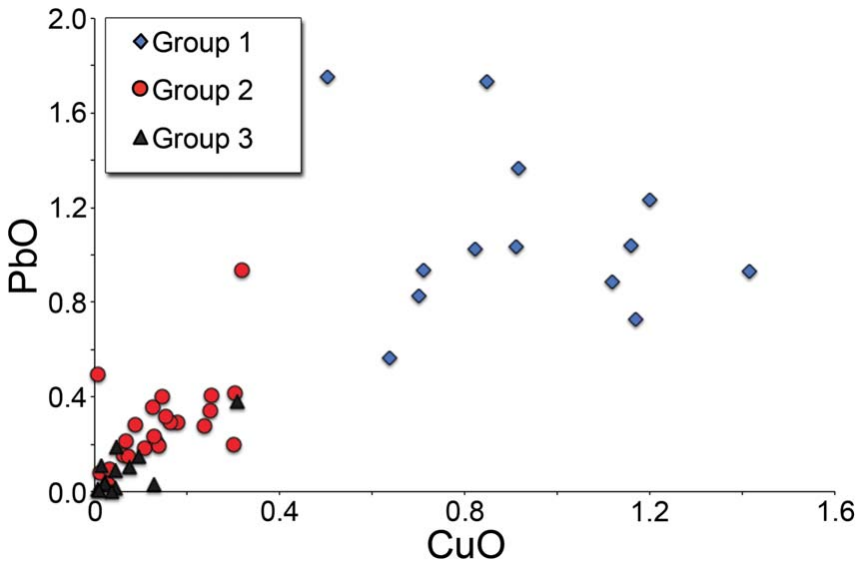
Colourless and weakly coloured glass groups from San Vincenzo. Lead and copper oxide concentrations of colourless and weakly coloured transparent glasses identify three distinct groups that reflect different stages in the glass production and recycling processes at San Vincenzo.

**Figure 6:**
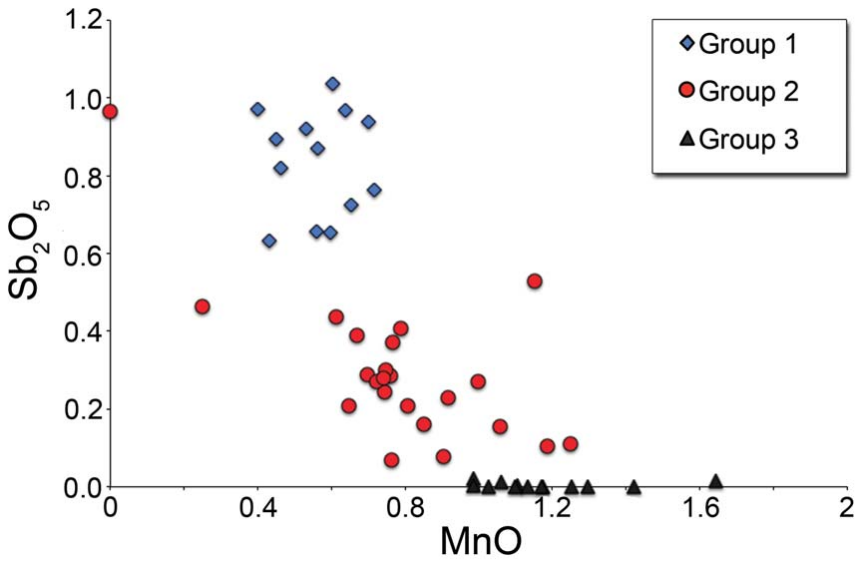
Antimony and manganese concentrations in colourless and weakly coloured glass. The three weakly coloured and colourless glass groups are differentiated. Group 3 with low antimony and high manganese does not appear to incorporate recycled glass.

The weakly coloured/colourless glasses fall into three groups on the basis of their minor element concentrations: Group 1 with >0.4% CuO, Group 3 with <0.05% Sb_2_O_5_, and an intermediate Group 2 (Figs. 5 and 6). It is noted that PbO and CuO are weakly positively correlated, while MnO and Sb_2_O_5_ are weakly negatively correlated. The three groups are distinguished in terms of their minor elements only. Silica, soda, lime and alumina concentrations are similar across all three groups. Groups 1 and 2 may be explained by the addition of material rich in Sb_2_O_5_, PbO and CuO to Group 3. Both FeO and K_2_O concentrations tend to be slightly higher in Group 1, while chlorine is lower, which can be explained by prolonged or repeated working of the glass.

Sample #44741-GAR (table 1) has MgO at 2.5% and K_2_O at 2.1%, levels generally considered to indicate the use of plant ash rather than natron as a source of soda. There is no evidence that such a composition was manipulated in the San Vincenzo workshops and this vessel appears to have been imported to the site.

## Discussion

### Coloured Glass

Glass production at San Vincenzo appears to have been strongly dependent upon the use of mosaic tesserae as sources of colour, sometimes used directly, as in the reticella canes which decorated some vessels, sometimes by partially mixing with less strongly coloured glass, as in the streaky red sheet glass, and sometimes by fully mixing and remelting, as in the cobalt-blue windows and vessels. Tessera compositions are typical of Roman mosaics of the first to third centuries and suggest that mosaics from old buildings were scavenged for raw materials. A few coloured glasses (for example translucent emerald green windows #44698, 44699) are not matched by tessera compositions analysed here. It is unclear at present whether these reflect manipulation of glass compositions by the addition of iron and/or copper oxides as colourants by the craftsmen of San Vincenzo or if they represent the use of Roman vessel glass. The production of greens using copper and iron was clearly within the Roman repertoire [34], although we are not aware of precise compositional parallels to the present examples from the Roman period. Irrespective of the origin of the translucent green glass and taking account of the full range of glass used on the site, it is clear that the abilities of the early medieval craftsmen to produce glass colours appear to have depended largely on the availability of old Roman material. Their skill to manipulate pigment raw materials to produce new colours appears to have been limited, relative to those of the craftsmen of earlier periods.

The use of cobalt-blue mosaic tesserae to colour window glass recalls the reference to the production of blue window glass in *De Rerum Artibus*, an account of medieval crafts by Theophilus, believed to be the monk Roger of Helmershausen who wrote in the early twelfth century [35]. Theophilus unequivocally states that blue tesserae from old Roman mosaics were mixed with colourless glass to make blue sheets for stained glass windows. Figure 4 allows the possibility that blue tesserae were mixed with colourless glass to make translucent blue for windows, but also that a simple mixture of blue tesserae of different tones was used. In fact, glass at San Vincenzo which is near colourless (Group 3, see below) typically has more than 1% MnO, whereas the blue tesserae and the blue windows have lower concentrations, at around 0.5%. The mixing of tessera with colourless glass to produce the blue windows would have produced around 0.8% MnO and therefore seems unlikely. Translucent blue vessel glass typically contains 0.7% CoO and 1.4% Sb_2_O_5_ (mean of 13 samples) and again is consistent with a mixture of mid- and dark blue tesserae.

The re-use of Roman opaque glass as a source of colour for windows at San Vincenzo clearly links early medieval practice with that of the twelfth century for the production of blue windows, as described by Theophilus. Furthermore, it has recently been suggested that red glass in twelfth- to fourteenth-century stained glass windows was also produced by a complex process involving the mixing of different batches of glass [36]. While differing in detail, the production of blue and the attempts to produce red translucent glass sheets by mixing opaque red tesserae with other colours to produce glass with red streaks may be seen as precursors to the development of coloured window glass technology in the high medieval period, which initially in the twelfth century seem to have been partly based upon the mixing of glasses to make strong colours, as well as the addition of colourant metal oxides directly to the glass melt. By 1400 these practices seem to have disappeared [36] but they provide linkage between the early and later window glass technologies and suggest that stained glass technology has its roots in the practices of the early medieval period.

### Colourless and weakly-coloured glass

The source of the colourless glass is less clear at first inspection but the subdivision into Groups 1–3 and their differences and similarities shed light on its probable origin. Enhanced colourant elements such as Cu, Pb and Sb in weakly-coloured transparent glass are now widely accepted as evidence of glass recycling in which coloured glass is incidentally included [37]–[40]. A particular increase in colourant elements such as lead and copper has been noted from the seventh to eighth centuries, for example in the assemblage from the Crypta Balbi in Rome, and this has been attributed to an increase in glass recycling [39]–[41]. Similar compositional characteristics in glass vessels from northern Italy have been attributed to the inclusion of mosaic tesserae in the assemblage of recycled glass [42], and the probable use of tesserae as colourants has been documented from Lorsch, Germany [43]. Given the evidence for the use of mosaic tesserae as raw materials in the workshops of San Vincenzo, it is likely that the enhanced concentrations of colourant elements seen in Groups 1 and 2 reflect the addition of tesserae into the melting pot.

**Figure 7:**
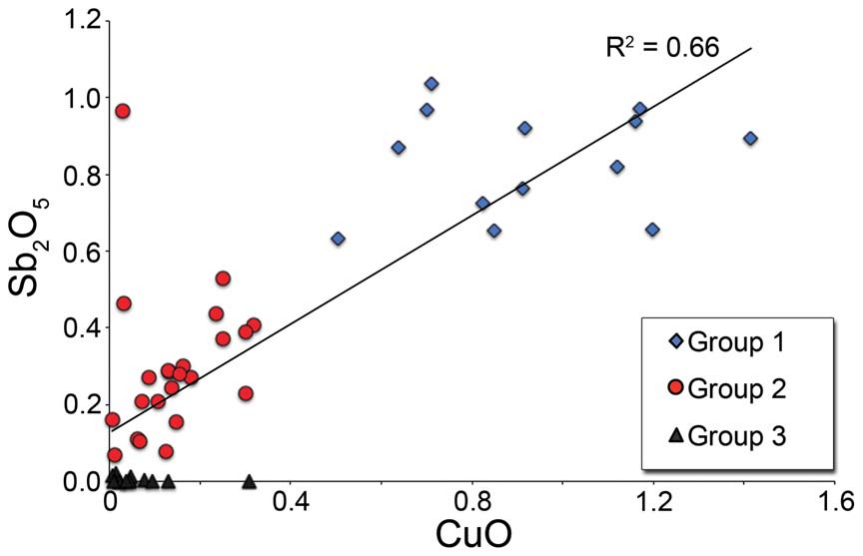
Correlation between copper and antimony oxides of the three glass groups from San Vincenzo. The positive correlation for all glasses (R^2^ = 0.66) indicates that the antimony contents of the San Vincenzo assemblage are not due to the incorporation of Roman antimony-decoloured glass, which has low copper.

Antimony could feasibly have been added due to the recycling of old Roman colourless glass which frequently contained antimony [38]. However, few San Vincenzo glasses have calcium levels as low as the antimony-decoloured glasses of the first to third centuries (Fig. 2). Furthermore, it seems improbable that antimony-decoloured glass makes a major contribution given the correlation seen between antimony and copper (Fig. 7), as copper is not commonly present at significant levels in Roman colourless glass. Indeed, only one analysed object has a composition closely resembling that of antimony-decoloured Roman glass from the first to third centuries, and that is sample #44758, a featureless vessel fragment which has the characteristic low-lime, low-alumina, and high soda (5.9% CaO, 1.8% Al_2_O_3_, 19.6% Na_2_O) composition typical of antimony-decolourised glass [38]. The sample is an outlier to the dataset in virtually all respects and was sampled for analysis because of its distinctive macroscopic appearance. It is an oddity in the assemblage and it is highly unlikely that glass of this composition was a major contributor to the bulk of the San Vincenzo glass.

The glass showing least evidence of recycling on the site is the colourless and weakly coloured transparent to translucent Group 3. This group has Sb_2_O_5_ below detection coupled with low or undetected CuO and PbO (Figs. 5 and 6). It contains approximately 1% MnO which was probably added as pyrolusite (MnO_2_) to oxidise the iron and decolour the glass [38], [44]. It is the closest to a primary glass of the samples analysed and is therefore likely to represent the colourless glass used as a raw material at San Vincenzo.

Manganese was used as a decolourant in soda-lime-silica glass from the second century BCE onwards [44]. Mn-decoloured glass was common in the first to third centuries CE, but also throughout most of the later first millennium CE, particularly in glasses which are currently believed to have been made in Egypt, where there are rich natural resources of manganese [45]. The large glass production centres of the eastern Mediterranean produced a number of characteristic compositional groupings, largely based on the composition of the sand used, which have been widely recognised (e.g. [5], [46]). These groups are all soda-lime-silica natron-type glasses, but differ in a number of respects, for example in their contents of MgO, FeO and TiO_2_, and lower or higher CaO and Al_2_O_3_. The standard lime-alumina plot of the major first millennium glass production groups (Fig. 2) underlines the difference between the glasses of San Vincenzo, including Group 3, and the main eastern Mediterranean glass groupings of the fourth to ninth centuries. This difference is emphasised when other components are considered. The Egyptian II grouping, which contains MnO at around the right level, has higher concentrations of TiO_2_ and lower K_2_O in addition to the significantly higher lime concentrations [47], while another Mn-rich group, HIMT [45] not shown in Fig. 2, has higher MgO and TiO_2_.

San Vincenzo Group 3 evidently differs from glass of the later first millennium (Fig. 2b), while it is remarkably similar to Roman manganese-decoloured glass of the first to third centuries (e.g. [38]). A comparison with a group of Mn-decoloured glass from a second century CE workshop in London [48] is given in Table 2. All oxides are within a single standard deviation in the two groups of glasses – they are essentially indistinguishable. Given the absence of parallels from the later first millennium, we conclude that the colourless glasses, like the coloured glasses at San Vincenzo, are based upon the re-use of old Roman glass from the first to third centuries.

**Table 2:** Comparison of the mean composition of San Vincenzo Group 3 with Mn-decoloured glass from a second century glass workshop at Basinghall Street, London [48].

	Group 3		London*	
	m (12)	s.d.	m (16)	s.d.
SiO_2_	67.94	*1.39*	69.83	*0.90*
Al_2_O_3_	2.40	*0.17*	2.24	*0.09*
FeO	0.54	*0.15*	0.38	*0.10*
CaO	7.32	*0.46*	7.72	*0.28*
MgO	0.65	*0.09*	0.54	*0.06*
Na_2_O	17.00	*1.09*	16.05	*0.85*
K_2_O	0.92	*0.55*	0.62	*0.10*
MnO	1.18	*0.20*	1.14	*0.20*
SO_3_	0.27	*0.12*	0.35	*0.11*
Cl	0.98	*0.14*	0.94	*0.20*

* The London glass was measured by SEM-EDXA and only elements at measurable concentrations in both groups are shown.

### Glass Production at San Vincenzo

Our data suggest several stages in the use of the glass raw materials at San Vincenzo. The first, represented by colourless Group 3, used unadulterated old Roman colourless glass to make vessels and windows. Coloured tesserae were used in the manufacture of reticella canes for the decoration of some of these vessels. For windows, tesserae were remelted and colours such as red and green were partially mixed to make streaky glass sheets, or fully mixed to make relatively homogeneous translucent blues for vessels and windows. A second stage, represented by glass Group 2, involved the production of weakly coloured glass by the mixing and melting of significant amounts of coloured opaque glass with colourless glass. Whether this was a direct addition of tesserae to the melting pot, or the recycling of waste production material from windows and decorated vessels is unclear. The variable compositions of the tesserae make it difficult to estimate the relative proportions of colourless and coloured glass in the batch at this stage. However, if it is assumed that the typical MnO content of the tesserae is about 0.5% and that of the Mn-decolourised glass is 1%, simple mass balance suggests that a continuum of up to 50% is very likely (Fig. 6). Group 2 clearly represents a number of melting events with varying mixtures of Mn-decolourised glass and tesserae. The nature of glass working activities suggests that recycling of production waste to make Group 2 compositions would inevitably follow a period of activity using the relatively pristine Group 3 glass to make vessels with coloured decoration, so the wide spread of compositions is readily understood.

Stage 3 is exceptional. The relatively low MnO and high Sb_2_O_5_, CuO and PbO in Group 1 glass (Figs. 5 and 6) can only be explained if this glass type was made mainly, if not entirely, from tesserae. Neglecting strongly cobalt blue glass, the addition of which would have imparted a strong colour and which was thus utilised specifically as a colourant for windows and vessels, the average composition of the mosaic tesserae analysed in this study includes 0.5% MnO, 0.9% CuO, 1.6% Sb_2_O_5_ and 1.2% PbO. The mean composition of Group 1 is 0.6% MnO, 0.9% CuO, 0.8% Sb_2_O_5_, 1.1% PbO. We are not able at this stage to suggest the relative proportions of the different opaque colours in the source mosaic (s) exploited at San Vincenzo. However, the potential to produce Group 1 from a batch comprising only mosaic tesserae is clear. While antimony in Group 1 is low compared to our average tessera composition, it is likely that the tessera average over-represents antimony opacified glass as this is widely assumed to have been more expensive and is therefore likely to have been less abundant in the mosaics than our simple average would suggest. White, for example, is likely to have been mainly obtained by the use of marble tesserae for the white elements of mosaics as indicated also by the failed attempt to melt marble tesserae at San Vincenzo (Fig. 1d). Allowing for this, and for the conservation by the glass workers of some heavily antimony-opacified colours which were used not only as trails on glass vessels but also in enamelwork on metal [49], then we may consider Group 1 to have been made largely, if not entirely, from mosaic tesserae.

The tendency for increased levels of K_2_O and FeO and low chloride observed in Group 1 is accounted for by increased potassium and iron contents in some tessera types (K and Fe in opaque red, Fe in yellow and black), while low chloride is a characteristic of Roman opaque white and turquoise glasses [50]. Furthermore potassium contamination from wood ash and fuel vapour is now widely recognised, from both experimental replication of Roman glassmaking practices [51] and from glass workshop debris [52]. The prolonged melting needed to homogenise opaque tesserae and colourless glass would have resulted in a higher concentration of K_2_O and conversely would also have tended to vaporise chlorine. Thus, all of the compositional differences between Groups 1, 2 and 3 may be explained by the differential recycling process. Furthermore the interrelations of a range of colourant elements suggest that a general mix of tesserae was being added, rather than specific colours.

### Trade and procurement of glass in the late first millennium CE

Glass used at San Vincenzo seems to have been based almost exclusively on old Roman colourless and opaque coloured glass that had been made some 500 years previously. We have detected only one fragment of a contemporary eighth to ninth century natron vessel in our investigation of the glass on the site (#44743-GAR, a featureless vessel shard related to the Egypt 2 compositional group), and one vessel (#44741-GAR) and one window sheet (#44728) possibly made of plant ash glass. This appears counter-intuitive as in the fourth to seventh centuries a supply of contemporary glass from the eastern Mediterranean reached even distant areas such as Britain [19], [53], [54]. However, compositional data from the Levant have been interpreted to suggest a decline in the availability of natron from as early as the fourth century [5], [55], [56] and it was no longer used in glass-making from the mid-ninth century.

While it is tempting to ascribe the total dependence upon old Roman glass at San Vincenzo to the decline in natron glass production in the east, this technological change does not, on the basis of our current information, appear to have caused a decline in the use of glass in the Levant and Egypt, where glass made from plant ashes appears to have immediately replaced glass made from natron. For example at Raqqa, Syria, natron glass and two varieties of plant ash glass have been in use more-or-less simultaneously around 800 CE [57]. However, we have only a single sample of possible plant ash-based glass that may have been worked at San Vincenzo and a single imported fragment.

An alternative explanation for the dependency upon recycled glass may therefore be the major downturn in East-West Mediterranean trade in the later first millennium which reached its nadir in the eighth to ninth centuries (as indicated for example by the production and distribution of pottery [58] and the apparent decline in the occurrence of Mediterranean shipwrecks [59]). This would suggest that fresh glass from the East may not have reached Italy because the trading network to carry it was greatly reduced. A further contributory factor is likely to have been that of import substitution: the glass workers in Italy did not need so much imported raw glass, because the glass from old buildings in the region provided what they needed. This in turn poses a further question: was the decline in the production of natron glass in the large tank furnaces on the Levantine coast and Egypt due to a decline or restriction in availability of a raw material (natron), as is generally assumed [13], [60]? Or was it due to the restricted nature of the market for this material, encouraging the development of a new production system, more flexible and immediately responsive to local or regional demand and based upon a more readily available raw material (plant ash)?

Whatever the reason for the lack of fresh glass from the East, the question remains as to how the monastery of San Vincenzo obtained its glass for recycling. Most publications are vague on the subject of just how glass was recycled but our data allow us to be quite specific for the present case. The idea that there existed a large reservoir of glass initially built up in the early imperial period and repeatedly recycled, with additions of fresh glass, over the fourth to eighth centuries is not viable for the simple reason that the predominant compositions here are specifically those of glass from the first to third centuries. The San Vincenzo assemblage does not show significant evidence of additions of glass types from the fourth century and later, which were richer in components such as Al, Fe and Ti for colourless glass and Sn for coloured glass. Hence, the idea that the San Vincenzo glass was obtained from a pool of glass which had been continuously recycled since Roman times can be ruled out with some certainty.

Another model for the procurement of colourless glass includes the possibility of widespread scavenging and collection of material from old buildings. However, the limited compositional range of the Mn-decolourised glasses of San Vincenzo (Group 3) and the absence of significant concentrations of antimony from this group strongly suggest that this practice was not the main source of old glass either. Weakly coloured blue-green glass, the most common Roman variety, contains both antimony and manganese, while a significant proportion of colourless glass was decoloured using antimony [38], [61]. While we have identified a few samples of glass that may represent material collected by scavenging, the Group 3 glass composition as a whole does not reflect the presence of significant amounts of these other Roman glass types. Group 3 is very consistent in composition, suggesting a single source. The most obvious source for this material is the window glass from a large Roman building. The tessera compositions may in turn have been derived from the mosaics of a single building also, although this is more difficult to demonstrate with any degree of certainty.

The re-use of Roman glass and tessera in early medieval glass production has been inferred for some time, due to the widespread archaeological association of tesserae with glass production and enamelling remains [62]. However, the particular contribution of the present study is the clear identification of the colourless glass from the site and its attribution to the window glass of a single Roman building. This is consistent with other lines of evidence. Columns of Egyptian (Aswan) granite are present at San Vincenzo, and these ultimately must have come from a large Roman public building. This is further substantiated by the *Chronicon Vulturnense*, an account of the history of the monastery by John, an abbot of the twelfth century, which records that San Vincenzo received a gift of a Roman temple in Capua (some 60 km away as the crow flies) and that the columns were used in the construction of the ninth-century church of *San Vincenzo Maggiore* [6]. The amount of glass included in some large public buildings of the Roman period is graphically illustrated by DeLaine's [63] architectural archaeology of the Baths of Caracalla in Rome. She estimates that this building contained 16,900 m^2^ of glass wall and vault mosaics and 3,400 m^2^ of window glass. The inferred 300 tonnes or so of vitreous materials have disappeared in the intervening centuries, presumably recycled into the melting pots of later generations. An interesting feature of the Baths of Caracalla is the preponderance of mosaic over window glass, as appears to have been the case in the building exploited by the glassworkers of San Vincenzo.

The conclusion that the San Vincenzo glass was obtained from the windows and mosaics of a single old Roman building, perhaps the same building as that from which the columns were obtained, is therefore consistent with other materials on the site, and with what we know of the use of glass in Roman buildings and the use of spolia in general in ninth-century building campaigns in Italy. It presupposes that Roman buildings still standing in the early ninth century retained some of their mosaic and window glass, but Theophilus implied that this was the case for mosaics even as late as the twelfth century [35]. The small scale of glassmaking in Italy in the preceding period and the effort needed to recover glass from the vaults of monumental buildings suggests that glass is unlikely to have been completely stripped from such buildings in a casual way and that major campaigns of recovery would have been needed to remove all of the material. An intriguing aspect of this study is therefore that it may provide insights into the condition of old Roman buildings in the landscape of the period, perhaps with substantial windows and mosaic-work still intact.

## Conclusions

The present data for the early ninth-century glass at San Vincenzo represent the most comprehensive study to date of glass compositions from a monastic complex of the Carolingian Renaissance. The glasses on the site are low in magnesia and potash and were made from natron, in the Roman tradition. Old mosaic tesserae, rich in antimony-based opacifiers, were used as sources of colour. Some weakly coloured transparent glass was produced by melting tesserae directly, but some glass represents the direct use of Roman glass decolourised by manganese. There is little or no indication that primary glass from the fourth to ninth centuries was incorporated into the San Vincenzo material on a large scale, suggesting that the early ninth-century glass workshops appear to have been almost totally dependent upon Roman glass from the first to third centuries.

By this time, fresh natron glass from the primary furnaces of the south-eastern Mediterranean appears to have been no longer accessible. However, the near-absence of eighth- to ninth-century primary natron glass compositions as well as high-magnesia plant ash glass suggests that it may have been the downturn in long-distance trade at this time, rather than a shortfall in the availability of natron, that restricted the supply of glass to San Vincenzo. This observation raises the possibility that the reduced distribution network for natron glass, effectively reducing the market for glass from the primary furnaces, contributed to its demise.

The limited compositional range of the colourless glass at San Vincenzo (almost all Mn- decoloured and containing only limited Sb) suggests that this represents a single group of material and that all of the glass from San Vincenzo may have been obtained from the mosaics and windows of a single large Roman building, perhaps the “temple” said by a twelfth century chronicler to have been given to the monastery as a repository for building materials. Investigations of similar assemblages of glass are required to determine if this is likely to be the case elsewhere and, furthermore, investigations of the variability of the architectural glass associated with large Roman buildings are desirable to test our assumptions. If correct, it implies that glass making activities at San Vincenzo were underpinned by patronage, in the form of elite gift-giving, rather than by a market economy. This in turn fed a craft industry producing luxury items for display, for the benefit of the religious elite and their guests [7].

The ability of the San Vincenzo craftsmen to manipulate glass raw materials, colourants and opacifiers appears to have been limited. Connections with specialised sources of colour, such as cobalt, antimony and manganese, were long gone and the glass colours had been prepared centuries before. We have identified only a single colour (emerald green) with a composition that we cannot easily parallel in Roman glass and that may have been the result of adding copper and iron directly to a glass melt in the ninth century; however, the status of this must be considered uncertain. Mixing and melting of glasses of different colours appears to be the distinctive characteristic of glass production at this time and provides a potential link to the production technology of the stained glass windows of the twelfth century.

The extent to which the practices inferred here reflect eighth- to ninth-century glass production practice in general, as opposed to the circumstances of a particular monastery, is not yet fully clear. Furthermore, the degree to which the pattern seen here ultimately reflects a reduction in demand for fresh glass from the East due to the availability of a regionally available substitute material, as opposed to a restriction in supply, remains to be seen. While transparent glasses with high copper, lead and antimony contents and typical Roman lime and alumina concentrations are widespread towards the end of the first millennium, not only in Italy [39]–[41], [53] but also in Britain (e.g. Hamwic [64]) and Germany [2], [43], detailed re-analysis and/or extension of these datasets is required. However, as we have shown, the composition and production of primary glass is now sufficiently understood to allow a more sophisticated analysis of the recycling and re-use of old glass in the early medieval period than has previously been the case.
